# Benchmarking Safety Indicators of Surgical Treatment of Brain Metastases Combined with Intraoperative Radiotherapy: Results of Prospective Observational Study with Comparative Matched-Pair Analysis

**DOI:** 10.3390/cancers14061515

**Published:** 2022-03-16

**Authors:** Motaz Hamed, Anna-Laura Potthoff, Julian P. Layer, David Koch, Valeri Borger, Muriel Heimann, Davide Scafa, Gustavo R. Sarria, Jasmin A. Holz, Frederic Carsten Schmeel, Alexander Radbruch, Erdem Güresir, Niklas Schäfer, Patrick Schuss, Stephan Garbe, Frank A. Giordano, Ulrich Herrlinger, Hartmut Vatter, Leonard Christopher Schmeel, Matthias Schneider

**Affiliations:** 1 Department of Neurosurgery, University Hospital Bonn, 53127 Bonn, Germany; motaz.hamed@ukbonn.de (M.H.); valeri.borger@ukbonn.de (V.B.); muriel.heimann@ukbonn.de (M.H.); erdem.gueresir@ukbonn.de (E.G.); patrick.schuss@ukbonn.de (P.S.); hartmut.vatter@ukbonn.de (H.V.); 2 Department of Radiation Oncology, University Hospital Bonn, 53127 Bonn, Germany; julian.layer@ukbonn.de (J.P.L.); david.koch@ukbonn.de (D.K.); davide.scafa@ukbonn.de (D.S.); gustavo.sarria@ukbonn.de (G.R.S.); jasmin.holz@ukbonn.de (J.A.H.); stephan.garbe@ukbonn.de (S.G.); frank.giordano@ukbonn.de (F.A.G.); christopher.schmeel@ukbonn.de (L.C.S.); 3 Department of Neuroradiology, University Hospital Bonn, 53127 Bonn, Germany; carsten.schmeel@ukbonn.de (F.C.S.); alexander.radbruch@ukbonn.de (A.R.); 4 Division of Clinical Neuro-Oncology, Department of Neurology, University Hospital Bonn, 53127 Bonn, Germany; niklas.schaefer@ukbonn.de (N.S.); ulrich.herrlinger@ukbonn.de (U.H.); 5 Department of Neurosurgery, BG Klinikum Unfallkrankenhaus Berlin, 12683 Berlin, Germany

**Keywords:** surgery for brain metastasis, intraoperative radiotherapy, perioperative complication profiling

## Abstract

**Simple Summary:**

Patients with brain metastasis (BM) are at advanced stages of metastatic cancer, and surgical resection is often required in order to avoid severe neurologic deficits. After surgery, patients are usually committed to postoperative radiotherapy. In recent years, intraoperative radiotherapy (IORT) has been proposed as an alternative to conventional postsurgical radiation approaches. This possibility has several advantages, e.g., as IORT is administered only once during the surgical procedure, patients do not have to attend several radiotherapy sessions afterward. However, the application of radiation therapy directly into the open brain during surgery might be accompanied by severe perioperative complications and, therefore, might negatively impact the overall benefit. In the present study, we show that patients who underwent surgery for BM combined with IORT do not suffer from elevated levels of perioperative complications compared to patients without IORT. Therefore, IORT constitutes a safe treatment strategy for cancer patients with BM.

**Abstract:**

Intraoperative radiotherapy (IORT) of the operative cavity for surgically treated brain metastasis (BM) has gained increasing prominence with respect to improved local tumor control. However, IORT immediately performed at the time of surgery might be associated with increased levels of perioperative adverse events (PAEs). In the present study, we performed safety metric profiling in patients who had undergone surgery for BM with and without IORT in order to comparatively analyze feasibility of IORT as an adjuvant radiation approach. Between November 2020 and October 2021, 35 patients were surgically treated for BM with IORT at our neuro-oncological center. Perioperative complication profiles were collected in a prospective observational cohort study by means of patient safety indicators (PSIs), hospital-acquired conditions (HACs), and specific cranial-surgery-related complications (CSCs) as high-standard quality metric tools and compared to those of an institutional cohort of 388 patients with BM resection without IORT in a balanced comparative matched-pair analysis. Overall, 4 out of 35 patients (11%) with IORT in the course BM resection suffered from PAEs, accounting for 3 PSIs (9%) and 1 HAC (3%). Balanced matched-pair analysis did not reveal significant differences in the perioperative complication profiles between the cohorts of patients with and without IORT (*p* = 0.44). Thirty-day mortality rates were 6% for patients with IORT versus 8% for patients without IORT (*p* = 0.73). The present study demonstrates that IORT constitutes a safe and clinically feasible adjuvant treatment modality in patients undergoing surgical resection of BM.

## 1. Introduction

Postoperative adjuvant cranial radiotherapy constitutes the standard clinical practice following surgical resection of brain metastases (BMs) [1,2]. In recent years, low-energy X-ray intraoperative radiation therapy (IORT) has been increasingly proposed as a viable alternative to conventional postsurgical irradiation approaches, as this technique yields considerable advantages: the delay between surgery and radiation therapy is greatly reduced, the dose gradient is steep, and challenges in target volume delineation are avoided due to postoperative anatomical alterations. The combination of these beneficial factors is associated with reduced doses to healthy brain tissue and may prevent repopulation of residual microscopic disease [3,4,5,6,7]. The safety of IORT has been demonstrated in the setting of surgically resected glioblastoma [8] and its efficacy is currently being evaluated in a Phase III clinical trial [9]. To date, known data regarding IORT in surgically treated BM are limited to several single institutional studies indicating promising high rates of local tumor control and a low incidence of radiation necrosis [10]. These studies are mainly focused on radiotherapeutic parameters, such as dosage, size of applicator, and time span of IORT procedures [3,10,11]. From an interdisciplinary perspective, the avoidance of postoperative complications in patients with surgically resected BM is of paramount importance to prevent prolonging the initiation of further adjuvant (including systemic) therapies [12,13]. In the context of IORT in BM, it remains unknown whether the concentrated radiation exposure administered intraoperatively has adverse consequences, e.g., for wound healing or other identifiable postoperative complications for the severely affected patients.

Therefore, the present prospective observational study aimed to investigate the frequency or occurrence of established perioperative adverse events (PAEs) during and after IORT in surgically treated BM. The results obtained were compared with data from patients without IORT who underwent standard postoperative adjuvant radiotherapy in order to benchmark the value and potential safety of IORT in patients with surgically treated BM.

## 2. Materials and Methods

### 2.1. Patients

Study data of all consecutive patients who were admitted to the Neurosurgical Department of the University Hospital Bonn and underwent surgical resection of BM and IORT between November 2020 and October 2021 were prospectively collected and managed using SPSS (version 25, IBM Corp., Armonk, NY, USA). Informed consent was obtained from the patients or the patients’ representatives. Information collected for each patient included sociodemographic characteristics, location of primary tumor, radiological and histopathological characteristics of both the tumor of primary origin and the intracranial metastatic lesion, and functional status at admission, among others. The comorbidity burden was objectified using the Charlson comorbidity index (CCI) [14]. The Karnofsky performance score (KPS) was used to classify the patients according to their functional status at admission. A stratification cut-off of 70 was chosen according to Péus et al. with regard to the patient’s ability to carry on their normal activities and work [15].

All patients received preoperative contrast-enhanced T1-weighted MRI providing 3D image guidance for both surgery and radiation treatment. For IORT, optic nerves, chiasma, and brain stem were identified preoperatively and intraoperatively as organs at risk (OAR), and applied doses were defined based on dose–depth profiles of the utilized spherical applicators ranging from 1.5 to 5 cm in diameter. IORT was delivered using INTRABEAM^®^ 600 (Carl Zeiss Meditec AG, Oberkochen, Germany) nominal 50 kV photons at an optimal dose of 30 Gy prescribed to the applicator surface. A decrease in the prescribed dose toward 24 Gy was acceptable in case of OAR doses exceeding the constraints of 12 Gy to the optical nerves and chiasma or 12.5 Gy to the brain stem following the Quantitative Analyses of Normal Tissue Effects in the Clinic (QUANTEC) recommendations [16].

Perioperative complications were assessed by means of a publicly available list of events introduced by the Agency for Healthcare Research and Quality and the Center for Medicare and Medicaid Services, and referred to as patient safety indicators (PSIs) and hospital-acquired conditions (HACs) [17,18]. PSIs included the complicative occurrence of pressure ulcers, iatrogenic pneumothorax, transfusion reactions, peri- and postoperative hemorrhage, pulmonary embolism, acute postoperative respiratory failure, deep-vein thrombosis, postoperative sepsis, wound dehiscence, and accidental puncture or laceration. Within the group of HACs, screening was performed for pneumonia, catheter-associated urinary tract infections, blood incompatibility, crushing injury, manifestation of poor glycemic control (diabetic ketoacidosis, nonketotic hyperosmolar coma, and hyperglycemic coma), fall injuries, and vascular catheter-associated infections. In addition, to assess complications specific to cranial surgeries, postoperative periods were screened for iatrogenic postoperative infarction, cerebrospinal fluid (CSF) leakage, postoperative meningitis and ventriculitis, brain edema, cerebrovascular venous thrombosis, postoperative seizures, and postoperative new or worsened neurological deficits; they were classified as cranial-surgery-related complications (CSCs) as previously described [19,20]. Perioperative complications were defined as any intra- and/or postoperative adverse event with or without further surgical interventions occurring within 30 days of the initial BM resection.

This study was conducted in accordance with the Declaration of Helsinki and approved by the Ethics Committee of the University Hospital Bonn (approval number 018/21).

### 2.2. Matching Procedure

Matching was used to control for measured pretreatment variables that were prognostic of the outcome. For the matched-pair analysis, the statistical computing program R (version 4.1.2 (The R Foundation, Boston, MA, USA); The R Foundation for Statistical Computing, https://www.r-studio.com (accessed on 25 January 2022)) was used. Propensity score matching was performed at a ratio of 1:3 between the cohort of 35 prospectively collected patients with IORT and a retrospectively collected cohort of 388 patients with surgically treated BM without IORT. The matching cohort of patients without IORT consisted of all patients aged ≥18 years who had undergone surgery for BM at our neuro-oncological center between 2013 and 2018. Within this time span, no patient received IORT at our neuro-oncological center. The study protocol for retrospective data collection was approved by the local ethics committee (approval number 250/19). Informed consent was not sought as a retrospective study design was chosen. Patients with leptomeningeal disease were excluded. To produce a covariate balance in the two groups and therefore increase the robustness of the data, the following known prognostic parameters were selected for matching: age [21], KPS and CCI at admission [19,21,22], tumor entity, and status of solitary versus multiple BM [19]. The balance was measured by the standardized mean differences, variance ratios, and empirical cumulative density function statistics, and visualized using a love plot. To display the distribution of propensity scores, we chose a jitter plot.

### 2.3. Statistics

Data analyses were performed using the computer software package SPSS (version 25, IBM Corp., Armonk, NY, USA) and GraphPad PRISM (GraphPad Software, San Diego, CA, USA). Categorical variables were analyzed in contingency tables using Fisher’s exact test. The Mann–Whitney U test was chosen to compare continuous variables as the data were mostly not normally distributed. Results with *p* < 0.05 were considered statistically significant.

## 3. Results

### 3.1. Patient Baseline Characteristics

Between November 2020 and October 2021, 35 patients underwent surgical therapy for BM combined with IORT at our neuro-oncological center. The median age was 63 years (IQR 54–71 years) including 18 women (51%) and 17 men (49%) (Table 1). The most common tumor entity of the intracranial metastatic lesion was NSCLC (60%), followed by melanoma (11%), and breast cancer (6%). Fifteen patients (43%) suffered from multiple BMs at the date of the operation. The median dose of IORT was 30 Gy (16–30 Gy). The median duration of IORT was 20 min (IQR 17–21 min). Overall, perioperative unfavorable events following resection of BM combined with IORT were present in 4 of 35 patients (11%). Two patients (6%) died within 30 days of surgery. The reasons for death were fulminant pulmonary embolism on day 15 following neurosurgical resection and sepsis from postoperative catheter-associated urinary tract infection. For further details on patient characteristics, see Table 1.

The functional status at discharge objectified by the KPS did not significantly differ for the groups with and without IORT (70 (60–90) vs. 80 (70–90), *p* = 0.52).

### 3.2. Perioperative Complications

Subclassification of intraoperative and early postoperative unfavorable events revealed three PSIs and one HAC (Table 2).

PSIs consisted of postoperative hemorrhage with the need for revision surgery, postoperative pulmonary embolism, and postoperative sepsis from urinary tract infection in 1 out of 35 patients (3%). HACs accounted for postoperative pneumonia in one subject (3%). No specific CSC was found.

### 3.3. Safety-Metric Profiling for IORT in a Comparative Matched-Pair Analysis

**Figure 1:**
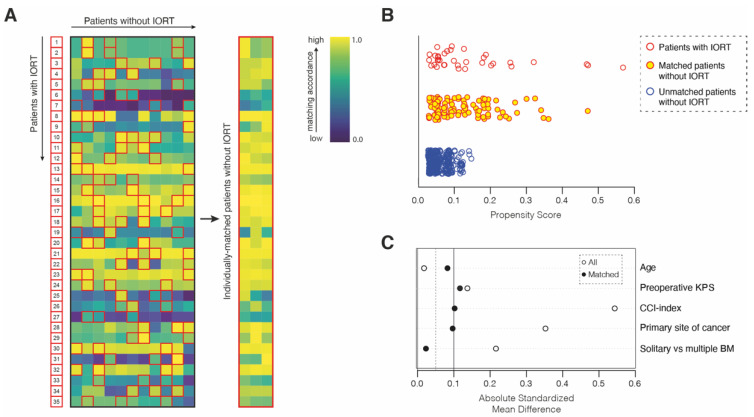
Illustration of the matching procedure for patients with surgically treated BM dependent on the presence of IORT. (**A**) Comparative matched-pair analysis at a ratio of 1:3 identified 105 out of 388 patients with surgically treated BM without IORT that individually corresponded to the present series of 35 patients with surgically treated BM combined with IORT. Heat map is a color-coded illustration of the matching strategy of patients without IORT to individually matched IORT cases by means of age at admission, KPS at admission, CCI at admission, and tumor entity and solitary versus multiple BM as matching parameters. Red frames depict individually matched patients without IORT. (**B**) Visualization of obtained propensity scores for matched and unmatched patients with BM. (**C**) Love plot depicting the balance of the matching analysis for each matching parameter determined by the standardized mean differences. BM, brain metastasis; CCI, Charlson comorbidity index; IORT, intraoperative radiotherapy; KPS, Karnofsky performance score.

In order to compare the complication risk profiles of patients with surgically treated BM dependent on the presence of IORT, we decided to perform multivariate and propensity score matching with additional balance optimization. For this purpose, patients with BM and IORT were individually matched at a ratio of 1:3 to a cohort of 388 patients that had undergone resection of BM without IORT between 2013 and 2018 at our neuro-oncological center (Figure 1).

Patient age, CCI and KPS at admission, primary site of cancer, as well as solitary versus multiple intracranial metastatic status as known prognostic parameters for survival in patients with an advanced metastatic stage of cancer disease were chosen as matching variables. Hence, the matched-pair analysis yielded two individually matched cohorts of 35 patients with IORT and 105 patients without IORT that did not significantly differ with regard to the above-mentioned prognostic survival parameters (Table 3). Safety-metric profiling by means of PSIs, HACs, and specific CSCs as highly standardized quality rating tools did not reveal significant differences between the two groups of matched patients with resected BM dependent on the presence of IORT (*p* = 0.44). Similarly, 30-day mortality did not significantly differ between patients with and without IORT (*p* = 0.73) (Table 3).

## 4. Discussion

IORT has gained growing attention with regard to its high rates of local tumor control as well as low incidences of radiation necrosis [10]. In addition, IORT should allow for the reduction of a patient’s socio-economic burden compared to standard surgery followed by multiple adjuvant radiation sessions [23], thereby also enabling faster initiation of systemic treatment. However, such additional intraoperative procedures accompanied by an extended time of surgery might result in elevated levels of early postoperative unfavorable events, which in turn may impair the initially supposed overall benefit of a combined surgery–radiation approach. In the present study, we aimed to analyze the prevalence of perioperative complications in patients who had undergone surgical removal of BM. The data indicate that the addition of IORT delivered immediately within the resection cavity following resection of BM is not associated with higher levels of perioperative unfavorable events within 30 days of surgery compared to a resection policy without IORT.

In contrast to the above-mentioned concerns of increased complication rates in the IORT cohort, these results might partly be attributed to radiation-induced activation of cell survival pathways and stimulation of the innate immune system. Both direct and indirect effects trigger inflammatory cytokine signaling and local immune cell recruitment [24], which may promote healing responses of the surgical-induced intracranial wound surface, leading to a superior antimicrobial milieu and reduced risk of intracavitary side infections. Compared to a postoperative stereotactic radiosurgical regime, the steep dose fall-off to the surgical wound surface in the setting of IORT results in lower dose exposure of the whole brain and in eloquent areas at risk, such as the optic apparatus and the brainstem [3]. Furthermore, by direct apposition of the applicator surface to the cavity wall, treatment dose delivery is highly accurate, and changes in cavity size due to tissue rearrangement during the delay between resection and radiation in case of SRS do not occur [10]. These advantages of IORT may have provided a rationale for the preservation of safety metric profiles of a conventional postoperative radiation regime observed in the present study.

In order to comprehensively assess the overall complication profiles, we used PSIs and HACs as publicly available and highly standardized classification tools [20]. Overall, the complication rate for the IORT group was found to reach about 11%. This level of PAE is within the range of reported data on the prevalence of perioperative complications in the course of BM surgery [25]. Moreover, a PSA- and HAC-based assessment of PAEs will also cover transient events, such as catheter-associated urinary tract infections and pneumonia and, therefore, quantitatively surpass previously reported complication levels of several studies that concentrated exclusively on adverse events that entailed further surgical interventions [26].

Thus, for the first time, the results of this study provide a benchmark insight into the expectable postoperative complication rate after BM surgery with simultaneous IORT and might thereby assist the consideration of potential benefits of such an approach for the individual patient. Beyond these insights into the feasibility of IORT as a modality of adjuvant radiotherapy, future analysis will have to comprehensively evaluate the issue of local tumor control rates and OS compared to conventional postsurgical irradiation approaches. The first available data indicate a 1-year local control rate of 88% in a cohort of 54 patients with BM and IORT with a median dose of 30 Gy [10]. In another study, where a median marginal dose of 20 Gy was used, a 1-year control rate of 84% was reported [11] indicating a dose dependency on the tumor control rate [27]. With regard to the advantages of a single intraoperative delivery compared to repeated postsurgical sessions of radiation, currently available data on tumor control indicate a promising overall benefit of IORT in the setting of surgically treated BM.

## 5. Limitations

Despite the prospective observational design of the present study, several limitations have to be considered in its interpretation. Primarily, the prospective sample size of 35 patients is considered quite small. Nevertheless, employing a matched-pair approach might have outweighed some confounding factors when comparing the safety profiles to those of BM patients without IORT. Furthermore, the present feasibility analysis of IORT in surgery for BM does not take into account neurocognitive assessment, which is known to significantly add to valuable outcome analysis in the field of neuro-oncology. Despite these limitations in sample size, the findings of the present study may suffice to conceive further large-scale, cross-regional databases to accurately evaluate the safety, feasibility, and efficacy of IORT in the setting of surgery for BM.

## 6. Conclusions

The present study findings indicate that IORT is a safe and clinically feasible adjuvant treatment modality in patients undergoing surgical resection of BM. The preservation of perioperative standard safety-metric profiles known for surgical BM resection without IORT presented herein might constitute another aspect to establish immediate intraoperatively delivered adjuvant radiation as a promising treatment approach. Further studies are needed to comprehensively evaluate the efficacy and overall value of IORT in the long-term follow-up.

## Figures and Tables

**Table 1:** Baseline characteristics of patients with IORT.

Baseline Characteristics	*n*
Female sex	18 (51%)
Median age (IQR) (in years)	63 (54–71)
Primary site of cancer	
Lung	21 (60%)
Melanoma	4 (11%)
Breast	2 (6%)
Others	8 (23%)
Multiple BMs	15 (43%)
Preoperative KPS ≥ 70	25 (71%)
Median CCI (IQR)	10 (8–11)
ASA ≥ 3	24 (69%)
Median duration of surgery (IQR)	272 (187–250)
Median dose of IORT (in Gy)	30 (16–30)
Median duration of IORT (IQR) (in min)	20 (17–21)
Perioperative complications	4 (11%)
30-day mortality	2 (6%)

Values represent the absolute number of patients unless indicated otherwise. ASA, American Society of Anesthesiology physical status classification system; BM, brain metastasis; CCI, Charlson comorbidity index; Gy, gray; IORT, intraoperative radiotherapy; IQR, interquartile range; KPS, Karnofsky performance status.

**Table 2:** Overview of perioperative complications of patients with IORT.

No. of Patients with IORT	*n* = 35
No. of complications	4 (11)
PSIs	
Postoperative hemorrhage	1 (3)
Postoperative pulmonary embolism	1 (3)
Postoperative sepsis (urinary tract infection)	1 (3)
HACs	
Pneumonia	1 (3)
Specific CSCs	0 (0)

Values represent the number of patients unless indicated otherwise (%). CSCs, cranial surgery-related complications; HAC, hospital-acquired conditions; IORT, intraoperative radiotherapy; No., number; PSIs, patient safety indicators.

**Table 3:** Comparative matched-pair analysis on perioperative complication profiles for patients with versus without IORT.

Variables	Surgery with IORT *n* = 35	Surgery without IORT *n* = 105	*p*-Value
**Matching variables**			
Age (years)	63 (54–71) ^†^	65 (56–73) ^†^	0.69
Preoperative KPS	80 (60–90) ^†^	80 (70–90) ^†^	0.73
CCI	10 (8–11) ^†^	9 (8–11) ^†^	0.74
Primary site of cancer			0.70
Lung cancer	21 (60)	58 (55)	
Others	14 (40)	47 (45)	
Solitary versus multiple BM			1.0
Multiple BM	15 (43)	46 (44)	
Solitary BM	20 (57)	59 (56)	
**Perioperative complications**	4 (11)	19 (18)	0.44
PSIs	3 (9)	12 (11)	n.s.
HACs	1 (3)	3 (3)	n.s.
Specific CSCs	0 (0)	4 (4)	0.04
**30-day mortality**	2 (6)	8 (8%)	0.73

Values represent the number of patients unless indicated otherwise (%). BM, brain metastasis; CCI, Charlson comorbidity index; CSCs, cranial surgery-related complications; HAC, hospital-acquired conditions; IORT, intraoperative radiotherapy; IQR, interquartile range; KPS, Karnofsky performance status; PSIs, patient safety indicators. ^†^ Median (IQR).

## Data Availability

No new data were created or analyzed in this study. Data sharing is not applicable to this article.
